# Dietary Cassava Roots Modified by Heat‐Moisture Treatment Improve Performance, Glucose Metabolism, and Carcass Yield in Fattening Goats

**DOI:** 10.1155/vmi/1557007

**Published:** 2026-06-16

**Authors:** Tossaporn Incharoen, Suban Foiklang, Wilasinee Inyawilert, Punnares Rattanapradit, Wirot Likittrakulwong, Tossapol Moonmanee, Juan J. Loor, Norakamol Laorodphan

**Affiliations:** ^1^ Department of Agricultural Science, Faculty of Agriculture, Natural Resources and Environment, Naresuan University, Phitsanulok, 65000, Thailand, nu.ac.th; ^2^ Faculty of Animal Science and Technology, Maejo University, Chiang Mai, 50290, Thailand, mju.ac.th; ^3^ Faculty of Food and Agricultural Technology, Pibulsongkram Rajabhat University, Phitsanulok, 65000, Thailand, psru.ac.th; ^4^ Department of Animal and Aquatic Sciences, Faculty of Agriculture, Chiang Mai University, Chiang Mai, 50200, Thailand, cmu.ac.th; ^5^ Department of Animal Sciences, University of Illinois, Urbana, 61801, Illinois, USA, illinois.edu

**Keywords:** apparent nutrient digestibility, cassava roots, goat nutrition, growth performance, heat-moisture treatment, resistant starch

## Abstract

**Background:**

Heat‐moisture–treated cassava roots (HMC) offer a promising nutritional strategy to enhance energy supply and nutrient utilization in fattening goats, primarily by modifying the starch structure to increase the resistant starch content. However, a comprehensive evaluation of its effects on performance, metabolism, and meat quality remains insufficiently characterized. In this study, we aimed to evaluate the effect of HMC inclusion in goat diets on growth, apparent nutrient digestibility, ruminal ecology, blood biochemistry, carcass yield, and meat quality.

**Methods:**

Twenty‐four male crossbred Anglo‐Nubian goats were randomly divided into four groups and fed diets containing 0, 100, 200, or 300 g/kg dry matter HMC for 60 days. All treatments were isonitrogenous and included ad libitum access to Napier grass.

**Results:**

Dietary inclusion of 200 g/kg HMC increased the average daily gain and improved the feed‐to‐gain ratio without affecting total dry matter intake. The apparent nutrient digestibility was not significantly altered (*p* > 0.05) by the inclusion levels of HMC. Similarly, the ruminal pH remained stable across treatments. Blood glucose concentration declined with 200–300 g/kg HMC inclusion, whereas other biochemical indicators were unaltered. Although the proportion of lean meat was reduced, carcass dressing percentage improved with dietary 300 g/kg HMC. No differences were observed in chemical composition, color, shear force, or pH of meat among the groups.

**Conclusion:**

Moderate dietary inclusion of HMC, particularly 200 g/kg DM, enhanced growth and carcass yield in fattening goats without compromising meat quality. However, excessive use may reduce apparent protein digestibility and lean meat yield. Thus, HMC inclusion may serve as an effective nutritional strategy to optimize productivity in goat production systems. Future studies should investigate the applicability of these findings in sheep and other large ruminant species.

## 1. Introduction

In goat production, providing diets with optimal energy and starch concentrations is essential to meet their intense metabolic demands and maximize overall performance. Interestingly, research indicates that goats exhibit a unique metabolic capacity to efficiently partition energy derived from high‐starch diets toward productive outcomes, rather than excessive body fat accumulation [1]. Furthermore, the efficiency of starch utilization is significantly influenced by the site of digestion. Shifting starch fermentation from the rumen to enzymatic hydrolysis in the small intestine has been shown to increase systemic glucose availability, which directly stimulates skeletal muscle protein synthesis and enhances overall growth performance [2]. Among energy‐dense feedstuffs, cassava (*Manihot esculenta Crantz*) is a crucial agricultural crop worldwide, serving as both a staple and livestock feed. The carbohydrate content of cassava, predominantly starch, comprises approximately 80% of its dry matter (DM) [3]. The inclusion of starch‐rich plant materials in ruminant diets yields nutritional, environmental, and economic benefits conducive to sustainable livestock production. Enhanced ruminal fermentation efficiency from high‐starch diets elevates fermentable carbohydrate availability, typically increasing propionate production, a key glucogenic substrate in ruminants [4]. Starch enrichment has also been linked to improved milk quality in dairy goats, particularly when combined with biliary acid supplementation [5].

Despite its benefits, excessive consumption of starch in ruminant diets can lead to subacute ruminal acidosis (SARA), a condition marked by a decline in ruminal pH and disruptions in microbial activity. SARA occurs when there is a reduction in pH typically ranging from 5.5 to 5.0 for certain periods during the day, resulting in an imbalance in ruminal metabolism [6]. High‐starch diets influence the ruminal environment by altering the microbial composition and its metabolic processes. When ruminants consume large amounts of starchy grains without adequate adaptation, the rapid fermentation of carbohydrates produces excess short‐chain fatty acids (SCFAs) and lactate, leading to a decrease in pH [7]. Such conditions favor the growth of lactic acid‐producing bacteria, which further acidify the ruminal environment, thus damaging the ruminal epithelium [8]. Methane is a significant by‐product of anaerobic microbial fermentation in the rumen associated with the loss of potential energy that could be otherwise utilized by the animal. This process is influenced by the microbial population, which plays a pivotal role in fermentation and subsequent digestion [9]. Generally, postruminal starch fermentation is considered more energy efficient compared with ruminal fermentation. Starch that escapes ruminal fermentation is typically digested in the small intestine or fermented in the large intestine, where it can be more completely utilized [10]. Thus, dietary strategies aimed at reducing ruminal starch degradation and enhancing postruminal digestion are warranted.

Heat‐moisture treatment (HMT), causing physical modification of starch under limited moisture and elevated temperatures, disrupts the crystalline structures and double helices in starch polymers [11]. Subsequent low‐temperature storage facilitates starch recrystallization, forming resistant starch (RS) type 3, which resists amylolytic enzymes [12]. The HMT modifies the physicochemical properties of starch, increasing its resistance to ruminal degradation and altering its gelatinization [13]. Recent studies using nylon‐bag techniques have demonstrated that HMT cassava chips lowered soluble fractions and degradability parameters [14]. The RS‐enriched cassava decreases ruminal DM degradation, gas, volatile fatty acids, and methane emissions while increasing bacterial populations including *Streptococcus bovis* and *Bacteroides* [13]. These findings indicate that RS redirects a portion of dietary starch from digestion in the rumen to postruminal sites, including the intestines, thereby compensating for decreases in ruminal fermentation. Srakaew et al. [15] reported enhanced nutrient intake and growth performance in beef bulls fed cassava chips processed via heat‐moisture treatment. However, extrapolating these nutritional strategies from large to small ruminants requires caution due to their distinct ruminal retention times and metabolic adaptations. Recent evidence in goats demonstrates that modulating starch digestion yields profound benefits. Specifically, enhancing bypass starch through cassava modification successfully sustains growth and maintains optimal rumen fermentation [16]. Additionally, shifting starch digestion to the small intestine increases systemic glucose, directly stimulating skeletal muscle protein synthesis and overall growth [2].

Consequently, utilizing RS‐rich sources, such as heat‐moisture–treated cassava, represents a highly effective dietary strategy to optimize finishing outcomes in small ruminants. Based on this background, we hypothesized that the dietary inclusion of heat‐moisture–treated cassava roots (HMC) would enhance growth performance and nutrient utilization in fattening goats. Thus, this study aimed to evaluate effects of HMC on growth, apparent nutrient digestibility, ruminal ecology, carcass traits, meat quality, and blood biochemistry in fattening goats.

## 2. Materials and Methods

### 2.1. HMC Preparations

Fresh cassava roots were procured from local cassava farms in Phitsanulok province, Thailand. Roots were washed thoroughly and chopped using an electric forage chopper (Model 2233HS‐18, Patipong Industry Co., Ltd., Pathum Thani, Thailand; powered by a 10‐HP motor with a processing capacity of 2 tons/h). The chopped cassava was then placed in stainless‐steel trays, weighed, and mixed with water at 10% (w/w). The mixture was sealed with aluminum foil and incubated in a hot‐air oven at 70°C for 12 h. Following incubation, the material was removed from the foil, cooled for 2 h at room temperature, and dried in a hot‐air oven at 60 ± 5°C until the moisture content was below 12%. Lastly, the dried HMC was ground and passed through a 4‐mm sieve prior to feeding.

### 2.2. Animal Ethical Statement

All experimental procedures were conducted in accordance with the guidelines for the care and use of agricultural animals in research. The study was officially approved by the Institutional Animal Ethics Committee of Pibulsongkram (Approval No.: PSRU‐(AG)‐2020‐010).

### 2.3. Animals, Diets, and Management

Twenty‐four male crossbred Anglo‐Nubian goats (average body weight: 15 ± 2 kg; age: 8 months) were used in this study. Animals were randomly allocated to four groups consisting of six goats each, following a completely randomized design. A 14‐day adaptation phase was conducted before the experimental feeding period. The feeding trial lasted for 60 days. Milled (1.5‐mm sieve) HMC was included at levels of 0, 100, 200, and 300 g/kg DM, replacing cassava meal (Table 1). These graded levels were strategically chosen to establish a dose–response relationship, with the maximum level of 300 g/kg DM set as a safe upper limit to prevent potential ruminal acidosis and maintain optimal fiber digestion in fattening goats. All diets were formulated to be isonitrogenous (16.48% CP) based on the requirements of local indigenous goats (BW 15 kg; ADG 100–150 g/d), according to the NRC recommendations for goats [17].

**TABLE 1:** Ingredient proportion of the experimental diet and analyzed chemical composition of the feed and Napier grass.

Item	Napier grass	Dietary HMC level (g/kg DM)			
		0	100	200	300
Ingredient (g/kg DM)					
Cassava meal		300	200	100	0
Modified cassava		0	100	200	300
Ground corn		203	203	203	203
Palm kernel meal		148	148	148	148
Fine rice bran		49	49	49	49
Corn gluten feed		119	119	119	119
Soybean meal		97	97	97	97
Molasses		48	48	48	48
Urea		13	13	13	13
Vitamin–mineral premix^1^		4	4	4	4
Dicalcium phosphate		4	4	4	4
Sodium sulfate		1	1	1	1
Salt		4	4	4	4
Calcium carbonate		10	10	10	10
Total		1000	1000	1000	1000
Chemical composition (%)					
ME (kcal/kg DM)^2^		2897.75	2911.65	2925.45	2939.25
TDN		75.94	75.54	75.14	74.74
Crude protein	4.50	16.48	16.49	16.51	16.52
Dry matter	19.52	88.95	89.38	89.82	90.25
Crude fat	2.07	3.31	3.30	3.29	3.28
Crude fiber	37.33	6.55	6.57	6.60	6.63
Organic matter	93.60				
Neutral detergent fiber	83.23				
Acid detergent fiber	53.31				
Feed price (THB/kg)	1.20	10.49	10.57	10.74	10.93

*Note:* HMC = heat‐moisture–treated cassava roots.

Abbreviations: DM = dry matter; ME = metabolizable energy; TDN = total digestible nutrients.

^1^Vitamin‐mineral premix provided the following per kilogram of diet: Vitamin A, 3,000,000 IU; Vitamin D3, 600,000 IU; Vitamin *E*, 5000 IU; biotin, 0.008 g; Co, 0.32 g; Cu, 4.5 g; I, 0.95 g; Mn, 17 g; Zn, 10.4 g; Fe, 6.25 g; Mg, 5.5 g; P, 20 g; iron, 30 g; Se, 0.5 g.

^2^Calculated based on the equations provided by the National Research Council [17].

Prior to the commencement of the experiment, all goats underwent a standard quarantine and health adaptation period. During this time, the animals were screened and confirmed negative for Brucellosis. Furthermore, they were vaccinated against foot‐and‐mouth disease (FMD), hemorrhagic septicemia, and blackleg in accordance with local veterinary guidelines. All goats were also dewormed using albendazole to ensure optimal health and eliminate internal parasites before the feeding trial began. Goats were individually housed in pens measuring 1.4 m × 1.4 m with wooden slatted floors. The animals were fed experimental concentrate diets at 2% of their body weight on a DM basis, while chopped Napier grass was provided for ad libitum access. The total daily DM intake (DMI) for each animal was determined by combining the measured consumption of both the concentrate and the Napier grass. Diets were offered twice daily at 07:00 and 17:00. Clean freshwater and mineral blocks were used throughout the experiments. Feed refusals were collected daily and weighed, and a representative subsample was dried at 60°C until a constant weight was obtained before the proximate analysis. The DMI was calculated by subtracting the dry weight of the refusals from the dry weight of the offered feed. All goats were weighed individually on a single designated day before the morning feeding and watering (following an overnight withholding of feed and water) at the start of the trial and every 15 days. This strict timing was implemented to minimize body weight variability associated with gut fill. Growth performance parameters, including weight gain, average daily gain (ADG), and feed‐to‐gain ratio, were calculated during the 60‐day fattening period.

### 2.4. Measurements of Apparent Nutrient Digestibility and Ruminal Ecology

Before each daily feeding, feed offered, refusals, and fecal samples were collected individually from each goat during the last three consecutive days of the experimental period. Samples were immediately stored in sealed plastic containers and frozen at −20°C to prevent degradation until chemical analysis. Prior to analysis, samples were dried in a forced‐air oven at 60°C for 72 h and ground to pass through a 1‐mm sieve using a Cyclotec Mill (Tecator, Hoganas, Sweden). The DM, organic matter (OM), and crude protein (CP) contents were determined following AOAC [18] methods 924.05, 942.05, and 988.05, respectively. Neutral detergent fiber (NDF) and acid detergent fiber (ADF) were analyzed as described by Van Soest et al. [19]. Apparent nutrient digestibility coefficients were calculated using the following standard formula:

(1) apparent digestibility %=feed intake kg per day−fecal kg per dayfeed intake kg per day×100.

Ruminal fluid samples (approximately 100 mL per goat) were aseptically collected at two critical time points: immediately prior to feeding (0 h) and 4 h after feeding. Collection was performed using a flexible stomach tube connected to a vacuum pump to ensure efficient retrieval with minimal animal stress. To prevent salivary contamination, the initial fraction of ruminal fluid was discarded. The pH of freshly obtained ruminal fluid was promptly measured on‐site using a calibrated portable pH meter (HI‐83141–1, Hanna Instruments, Padova, Italy) to ensure the accurate assessment of ruminal acidity.

Subsequently, the samples were filtered through four layers of sterile cheesecloth to remove particulate matter and were transferred into sterile plastic containers. From the filtrate, 45‐mL aliquots were acidified immediately by adding 5 mL of 1 mol/L sulfuric acid to halt microbial activity and preserve the sample. The acidified samples were centrifuged at 16,000 × g for 15 min at 4°C to separate suspended solids. The resulting supernatant was analyzed for ammonia nitrogen (NH_3_–N) concentration using a Kjeltec Auto 1030 Analyzer (Foss Analytical, Hillerød, Denmark) following standard laboratory procedures.

### 2.5. Blood Biochemistry Determination

At the end of the feeding trial, blood samples were collected from each goat via jugular venipuncture using sterile 10‐mL vacutainer tubes without anticoagulant, following standardized animal welfare protocols. Blood samples (approximately 3 mL) were allowed to clot at room temperature for 30 min and subsequently centrifuged at 3000 × g for 15 min at 4°C to separate serum (FC5706, OHAUS Corporation, USA). The serum was carefully transferred into labeled Eppendorf tubes and immediately stored at −20°C until biochemical analysis. Concentrations of glucose, blood urea nitrogen (BUN), creatinine, uric acid, total bilirubin, cholesterol, total protein, albumin, aspartate aminotransferase (AST), and alanine aminotransferase (ALT) levels were determined using a VetTest Chemistry Analyzer (IDEXX Laboratories, Inc., Westbrook, ME, USA).

### 2.6. Carcass Characteristics and Meat Quality Analysis

Upon termination of the feeding experiment, all goats were slaughtered according to the ethical guidelines for animal care and use. Carcass characteristics were evaluated immediately after slaughter. The warm dressing percentage was calculated as the proportion of hot carcass weight to live body weight before evisceration. The primal cuts were then separated and weighed on a digital scale. The cuts included lean meat, forelegs, hind legs, briskets, tenderloins, sirloins, and necks.

For the chemical composition analysis, longissimus dorsi muscle samples were collected from each carcass. Proximate analysis was conducted to determine the DM, CP, and ether extract (EE) content using AOAC [18] procedures. Each meat sample was oven‐dried at 105°C, homogenized, and subjected to Kjeldahl nitrogen determination for CP and ether extraction for EE quantification. Muscle pH was measured 45 min postmortem by inserting the penetration probe of a portable pH meter (AG 8603, Mettler‐Toledo AG, Schwerzenbach, Switzerland) directly into the longissimus dorsi muscle. Calibration was performed using standard buffer solutions prior to measurement. All analyses were conducted in triplicate for accuracy.

Meat color was assessed within 45 min postmortem using a calibrated colorimeter (Minolta CR‐410, Konica Minolta, Japan). The color parameters L^∗^ (lightness), a^∗^ (redness), and b^∗^ (yellowness) were recorded from three random locations on the cut surface of the longissimus dorsi muscle and averaged per sample. The shear force was measured to assess meat tenderness. The cooked meat samples were prepared by heating the samples to an internal temperature of 70°C. After chilling, the samples were cut into 1 × 1 cm cross sections and analyzed using a texture analyzer (TA.XTplus, Stable Micro Systems, Godalming, Surrey, UK) equipped with a Warner‐Bratzler blade probe and operated using Texture Expert Exponent software (Stable Micro Systems Ltd., Godalming, Surrey, UK). The maximum force required to shear each sample was recorded in Newtons (N).

### 2.7. Statistical Analysis

The analyzed parameters encompassed growth performance, apparent nutrient digestibility, ruminal fermentation profiles, blood metabolites, and carcass and meat quality characteristics. Data collected from all treatments were analyzed by one‐way analysis of variance (ANOVA). The results are presented as mean values with the pooled standard error of the mean (pooled‐SEM). Differences among treatment means were determined using Tukey’s honestly significant difference (HSD) test, with statistical significance declared at *p* < 0.05. All analyses were performed using the Statistical Package for the Social Sciences (SPSS) software, Version 17 (SPSS Inc., Chicago, IL, USA).

## 3. Result

### 3.1. Growth Performance

The effects of dietary inclusion of HMC on the growth performance of fattening goats are presented in Table 2. The initial and final body weights did not differ (*p* > 0.05) among groups. Notably, in contrast to the control group, goats fed 200 g/kg HMC exhibited an improvement (*p* < 0.05) in growth metrics, achieving a total weight gain of 9.25 kg versus 5.63 kg and an ADG of 154.17 versus 93.83 g/d. Furthermore, as evidenced by a reduction (*p* = 0.039) in the feed‐to‐gain ratio from 8.91 in the controls to as low as 5.59 in the 200 g/kg group, the feed‐to‐gain ratio improved markedly at all HMC inclusion levels. Importantly, DMI was not (*p* = 0.967) affected by HMC inclusion, remaining consistent across all dietary groups (817.34–861.42 g/d), and indicating a lack of detrimental effect. These findings demonstrate that incorporating HMC into the diet at 200 g/kg DM enhances growth performance and feed efficiency without negatively affecting feed consumption in fattening goats, highlighting its potential as a valuable dietary strategy for small ruminant production systems.

**TABLE 2:** Growth performance of goats fed diets containing different levels of heat‐moisture–treated cassava roots (HMC).

Item	Dietary HMC level (g/kg DM)				SEM	*p* value
	0	100	200	300		
Growth performance						
Initial weight (kg)	17.25	17.13	16.75	15.75	1.15	0.974
Final weight (kg)	22.88	24.75	26.00	23.88	1.30	0.881
Weight gain (kg)	5.63^b^	7.62^ab^	9.25^a^	8.13^ab^	0.49	0.043
Total DMI (g/d)	835.75	830.07	861.42	817.34	28.67	0.967
ADG (g/d)	93.83^b^	127.00^ab^	154.17^a^	135.50^a^	8.14	0.043
F/G	8.91^b^	6.54^a^	5.59^a^	6.03^a^	7.03	0.039

*Note:* ^a,b^Values in the same row with different superscripts differ (*p* < 0.05).

Abbreviations: ADG = average daily gain; DMI = dry matter intake; F/G = feed‐to‐gain ratio; SEM = standard error of the mean.

### 3.2. Apparent Nutrient Digestibility and Ruminal Ecology

The effects of dietary HMC inclusion on apparent nutrient digestibility are presented in Table 3. The apparent digestibilities of DM and OM were not significantly altered (*p* > 0.05) by the inclusion levels of HMC. Similarly, the apparent digestibilities of CP, NDF, and ADF remained unaffected (*p* > 0.05) across all dietary treatments. Ruminal pH values, measured at baseline and postfeeding intervals, did not differ (*p* > 0.05) among treatments, maintaining a stable ruminal environment. These data indicate that moderate inclusion of HMC in goat diets can enhance protein utilization and alter nitrogen metabolism without disrupting ruminal pH homeostasis, suggesting beneficial effects on ruminal fermentation and overall nutrient assimilation.

**TABLE 3:** Apparent nutrient digestibility and ruminal ecology of goats fed diets containing different levels of heat‐moisture–treated cassava roots (HMC).

Item	Dietary HMC level (g/kg DM)				SEM	*p* value
	0	100	200	300		
Apparent digestibility (%)						
Dry matter	80.61	84.83	83.41	83.70	0.71	0.188
Organic matter	82.00	85.67	84.64	84.61	0.65	0.228
Crude protein	75.83	79.04	80.57	74.96	1.11	0.244
Neutral detergent fiber	83.46	86.55	83.08	85.03	0.61	0.158
Acid detergent fiber	71.37	77.60	75.19	77.02	1.21	0.276
Ruminal pH						
0 h	6.78	6.65	6.75	6.75	0.06	0.918
4 h	6.33	5.85	6.16	6.04	0.09	0.338
Ruminal NH_3_–N (mg/dL)						
0 h	13.83	15.75	18.55	15.40	1.05	0.496
4 h	14.00	16.28	21.18	13.30	1.35	0.149

Abbreviation: SEM = standard error of the mean.

### 3.3. Blood Biochemical Indices

The blood biochemical parameters of the goats fed varying levels of HMC are summarized in Table 4. A decrease (*p* = 0.001) in glucose concentration was evident with increasing HMC inclusion, with the highest glucose levels observed in the control group (55.20 mg/dL) and the lowest in the 300 g/kg group (39.80 mg/dL). No significant differences (*p* > 0.05) were detected in BUN, creatinine, uric acid, total bilirubin, cholesterol, total protein, albumin, AST, and ALT levels across treatments, indicating the absence of detrimental effects on renal function, liver integrity, and protein metabolism. These results suggest that while HMC inclusion modulates energy metabolism, as reflected by the reduction in blood glucose, it does not adversely influence overall metabolic health or organ function in fattening goats. This highlights the potential of HMC as a nutritionally safe feed ingredient in goat production systems.

**TABLE 4:** Serum biochemistry of goats fed diets containing different levels of heat‐moisture–treated cassava roots (HMC).

Item	Dietary HMC level (g/kg DM)				SEM	*p* value
	0	100	200	300		
Glucose (mg/dL)	55.20^b^	51.60^b^	43.20^a^	39.80^a^	1.82	0.001
BUN (mg/dL)	10.80	12.60	12.00	10.40	0.46	0.312
Creatinine (mg/dL)	0.61	0.64	0.66	0.62	0.03	0.953
Uric acid (mg/dL)	0.08	0.02	0.02	0.04	0.01	0.362
Total bilirubin (mg/dL)	0.10	0.10	0.14	0.12	0.01	0.261
Cholesterol (mg/dL)	53.00	51.80	73.20	60.20	4.39	0.305
Total protein (g/dL)	6.06	6.90	6.74	6.28	0.16	0.243
Albumin (g/dL)	2.58	2.94	3.00	2.86	0.08	0.310
AST (U/L)	69.00	68.20	72.40	76.20	1.95	0.484
ALT (U/L)	18.20	22.40	25.00	20.40	1.24	0.264

*Note:* ^a,b^Values in the same row with different superscripts differ (*p* < 0.05). The reference ranges for goats noted by Kaneko et al. [20] are as follows: glucose, 50.0–75.0 mg/dL; cholesterol, 80–130 mg/dL; blood urea nitrogen, 10.0–20.0 mg/dL; creatinine, 1.0–1.8 mg/dL; uric acid, 0.3–1.0 mg/dL; total protein, 6.4–7.0 g/dL; albumin, 2.7–3.9 g/dL; total bilirubin, 0.0–0.1 mg/dL; aspartate aminotransferase, 67–513 U/L; alanine aminotransferase, 6–19 U/L.

Abbreviations: ALT = alanine aminotransferase, AST = aspartate aminotransferase, BUN = blood urea nitrogen, SEM = standard error of the mean.

### 3.4. Carcass Characteristics and Meat Quality

The carcass characteristics and meat quality traits of goats fed diets containing varying levels of HMC are presented in Table 5. Compared with other treatments, the warm dressing percentage increased (52.67%; *p* = 0.002) with the 300 g/kg HMC inclusion rate, indicating an improved carcass yield. Conversely, the proportion of lean meat decreased (*p* = 0.001) with increasing HMC inclusion, with the control group having the highest lean meat percentage (39.86%) and the 300 g/kg group the lowest (32.87%). Notably, compared with the control, foreleg weights were also higher (*p* = 0.007) at 100 and 200 g/kg HMC levels, whereas other primal cuts did not differ. The chemical composition of the meat, including DM, EE, and CP, as well as meat color (L^∗^, a^∗^, b^∗^), shear force, and pH at 45 min postmortem, did not differ (*p* > 0.05) among the treatments. These findings suggest that while high levels of dietary HMC can enhance carcass yield, there may be trade‐offs in lean meat proportion. The lack of significant changes in meat chemical and physical quality attributes suggests that HMC supplementation does not compromise the meat quality of fattening goats.

**TABLE 5:** Carcass characteristics and meat quality of goats fed diets containing different levels of heat‐moisture–treated cassava roots (HMC).

Item	Dietary HMC level (g/kg DM)				SEM	*p* value
	0	100	200	300		
Carcass characteristics (%)						
Warm dressing	44.94^b^	44.45^b^	44.12^b^	52.67^a^	1.17	0.002
Lean meat	39.86^a^	37.15^ab^	35.29^ab^	32.87^b^	0.84	0.001
Fore legs	4.34^b^	6.90^a^	6.66^a^	4.94^b^	0.38	0.007
Hind legs	7.41	8.81	7.82	6.59	0.32	0.081
Brisket	2.82	2.95	2.71	2.87	0.13	0.954
Tenderloin	1.20	1.22	1.33	1.03	0.04	0.085
Sirloin	6.54	6.87	6.69	5.71	0.18	0.076
Neck	6.91	7.74	7.66	7.06	0.49	0.932
Chemical composition of meat (%)						
Dry matter	27.50	31.93	28.86	26.43	0.12	0.567
Ether extract	3.66	4.20	3.95	4.06	0.41	0.441
Crude protein	23.67	25.78	26.01	26.24	1.35	0.071
Meat color						
L–	42.91	41.27	42.03	44.18	0.43	0.069
a–	11.68	13.16	13.09	14.48	0.39	0.062
b–	6.21	5.26	5.18	6.45	0.30	0.340
Shear force (*N*)	37.40	46.57	50.89	46.03	4.21	0.775
pH 45 min	6.42	6.41	6.31	6.44	0.06	0.900

*Note:* ^a,b^Values in the same row with different superscripts differ (*p* < 0.05). Reference ranges for goat meat color reported by Dhanda et al. [21] are as follows: L–, 33.7–43.6; a–, 10.3–12.4; b–, 6.7–8.1. L– = lightness. a– = redness, b– = yellowness.

Abbreviation: SEM = standard error of the mean.

## 4. Discussion

In the present study, increasing the inclusion level of HMC up to 300 g/kg DM did not adversely affect the apparent digestibilities of DM, OM, CP, NDF, and ADF (*p* > 0.05). Concurrently, crucial ruminal fermentation parameters, notably ruminal pH and NH_3_–N concentrations, remained stable and within optimal physiological ranges for goats across all dietary treatments. The stability of ruminal pH is a critical factor in maintaining a balanced ruminal ecosystem, particularly for the survival and optimal fibrolytic activity of cellulolytic bacteria [22]. This favorable ruminal environment can be directly attributed to the modified starch structure in HMC. By forming a tightly packed crystalline lattice of *α*‐1,4 linked glucose (amylose), the RS fraction resists enzymatic cleavage, thereby effectively reducing the rate of rapid starch degradation in the rumen [23]. This controlled fermentation prevents sudden acid accumulation, allowing fiber digestion to proceed unimpeded. Consequently, the sustained digestibilities of NDF and ADF observed in our study strongly indicate that the dietary inclusion of HMC did not disrupt the ruminal microbial ecology, despite the high‐energy carbohydrate supplementation.

Furthermore, the lack of a negative impact on CP digestibility provides significant insights into the appropriateness of the processing conditions used for HMC in this experiment. It is widely recognized that severe thermal processing of carbohydrate‐rich feeds can induce excessive nonenzymatic browning, commonly known as the Maillard reaction. This reaction forms irreversible cross‐linkages between reducing sugars and the amino groups of amino acids, rendering the dietary protein indigestible in both the rumen and the lower gastrointestinal tract [13]. Additionally, excessive heat can cause severe starch gelatinization and retrogradation, creating complexes that tightly encapsulate proteins and limit the accessibility of proteolytic enzymes [24]. However, our digestibility results clearly demonstrate that the specific HMT applied to the cassava roots was highly optimal. It successfully altered the starch matrix to bypass ruminal fermentation without crossing the threshold into detrimental structural modifications. Therefore, the consistent CP and OM digestibilities confirm that HMC can be effectively utilized as a high‐density energy ingredient that delivers intact nutrients for postruminal absorption without compromising overall feed utilization.

Despite total DMI and apparent total tract nutrient digestibilities remaining comparable across all treatments, goats fed the 200 g/kg HMC diet exhibited a remarkable enhancement in growth performance, achieving the highest ADG and the most efficient feed‐to‐gain ratio. Since the total proportion of digested nutrients was similar, this superior growth highlights a fundamental shift in the site of digestion and a subsequent increase in the metabolic efficiency of the absorbed end‐products. By shifting starch digestion postruminally, the mandatory energy losses typically associated with ruminal microbial metabolism, such as the heat of fermentation and methanogenesis, are substantially minimized [23]. Instead of being entirely converted into VFAs, the bypassed starch reaches the small intestine, where it is enzymatically hydrolyzed and absorbed directly as glucose [25]. When small intestinal starch digestibility exceeds 75%, glucose absorption in the small intestine demonstrates greater energetic efficiency than ruminal VFA production, offering improved nutrient utilization efficiency for the animal [26]. Energetically, starch digested and absorbed as glucose in the small intestine is approximately 25%–30% more efficient than ruminally fermented starch [27]. Therefore, this improved nutrient partitioning and elevated energetic efficiency primarily explain why the HMC‐supplemented goats achieved optimal growth without requiring a concurrent increase in total nutrient digestibility.

Regarding serum biochemistry, circulating blood glucose linearly decreased as the HMC inclusion level increased. This phenomenon relates to the unique carbohydrate metabolism in ruminants, which relies primarily on hepatic gluconeogenesis from ruminal propionate rather than direct intestinal glucose absorption [28]. Increasing HMC elevates bypass starch and reduces ruminal starch fermentation, consequently limiting the production of propionate. As propionate is the primary glucogenic precursor, its reduction directly limits hepatic glucose synthesis. Because physiological constraints restrict the extent of direct glucose absorption in the small intestine, this limited postruminal supply cannot fully compensate for the reduced hepatic output, resulting in lower systemic blood glucose. Despite this reduction, goats fed HMC diets did not experience an energy deficit due to the overall enhanced energetic efficiency provided by postruminal starch digestion. Furthermore, BUN concentrations remained unaffected (*p* > 0.05), confirming that HMC treatments did not impair protein metabolism or disrupt the ruminal energy‐to‐nitrogen balance.

A notable finding in this study was the concurrent increase in the warm dressing percentage and the decrease in the proportion of lean meat at the highest HMC inclusion level (300 g/kg), presenting a distinct shift in carcass yield dynamics. The abundant net energy derived from the improved energetic efficiency of bypass starch successfully drove greater overall carcass development and mass. However, muscle protein accretion is biologically constrained by the genetic potential of the animal [29]. Once lean tissue growth reaches its physiological plateau, any continuous increase in total carcass weight driven by surplus energy inherently dilutes the relative proportion of lean meat. This explains the observed reduction in lean percentage without implying actual muscle degradation or loss. This concept of relative dilution is strongly supported by the unaltered chemical composition of the meat, including CP and EE, across all treatments. Collectively, these findings indicate that while high‐bypass starch supplementation maximizes overall carcass yield, it alters carcass proportions without compromising the inherently stable physical and chemical meat quality of fattening goats.

## 5. Conclusion

Inclusion of HMC at 200 g/kg DM significantly enhances growth performance and carcass yield in fattening goats without adverse effects on total DMI and meat quality. While higher inclusion levels improve dressing percentage, they reduce the lean meat proportion, indicating a shift in nutrient partitioning where increased total carcass mass dilutes the relative muscle proportion. Blood biochemical markers confirm the metabolic safety of HMC. Overall, moderate inclusion rates of HMC offer a valuable nutritional strategy to optimize productivity in commercial goat production systems. However, further research should focus on balancing dietary protein–energy ratios to maximize meat quality and yield.

## Author Contributions

Tossaporn Incharoen: conceptualization, subversion, data curation, software, writing–original draft, and writing–review and editing. Wirot Likittrakulwong: formal analysis and software. Punnares Rattanapradit: data curation and software. Suban Foiklang: formal analysis and visualization. Wilasinee Inyawilert: formal analysis and data curation. Juan J. Loor: visualization and writing–review and editing. Norakamol Laorodphan: conceptualization, data curation, formal analysis, software, and writing–original draft.

## Funding

This study was funded by the National Research Council of Thailand (Grant No. 851/2563). Additional partial support was provided by the Frontier Research and Innovation Cluster Fund, Naresuan University (Grant No. R2569C002).

## Ethics Statement

The experimental protocol was reviewed and approved by the Animal Ethics Committee of Pibulsongkram Rajabhat University (Ethics ID: PSRU‐(AG)‐2020‐010), in accordance with the Ethical Guidelines for Animal Experimentation established by the National Research Council of Thailand. All procedures complied with internationally recognized guidelines for the care and use of animals in the study.

## Conflicts of Interest

The authors declare no conflicts of interest.

## Data Availability

The data that support the findings of this study are available from the corresponding author upon reasonable request.
